# Sociodemographic and Regional Differences in Cigarette Consumption Across Great Britain: A Population Study, 2022–2024

**DOI:** 10.1093/ntr/ntaf133

**Published:** 2025-07-18

**Authors:** Sarah E Jackson, Jamie Brown, Vera Buss, Sharon Cox

**Affiliations:** Department of Behavioural Science and Health, University College London, London, UK; Department of Behavioural Science and Health, University College London, London, UK; Behavioural Research UK, Edinburgh, UK; Department of Behavioural Science and Health, University College London, London, UK; Behavioural Research UK, Edinburgh, UK; Department of Behavioural Science and Health, University College London, London, UK; Behavioural Research UK, Edinburgh, UK

## Abstract

**Introduction:**

The harms of cigarette smoking are greater for those who smoke more heavily. This study aimed to provide up-to-date estimates of cigarette consumption across Great Britain and explore differences by socioeconomic position, the presence of children in the household, nation, and region.

**Methods:**

Cross-sectional analysis of data from a nationally representative household survey of adults in Great Britain, 2022–2024 (*n* = 77 796). Main outcome measures were mean daily cigarette consumption and the proportion consuming more than 20 cigarettes per day (among those who smoked cigarettes) and average per-capita cigarette consumption (among adults).

**Results:**

Overall, adults who smoked cigarettes consumed on average 10.4 [95%CI = 10.2%–10.6%] cigarettes per day, with 5.5% [5.0%–6.0%] smoking more than 20 per day. Average per-capita consumption was 528 [512–543] cigarettes per year, equating to a total consumption of 28.6 [27.8–29.5] billion cigarettes in Great Britain per year. Consumption was lower among those from more vs. less advantaged socioeconomic positions (mean consumption: 9.4 [9.1–9.7] vs. 11.0 [10.7–11.3] cigarettes per day; proportion smoking more than 20 per day: 4.6% [3.9%–5.2%] vs. 6.1% [5.4%–6.9%]). It was also lower among those with children in the household than those without (9.7 [9.3–10.0] vs. 10.7 [10.4–10.9]; 4.0% [3.2%–4.9%] vs. 6.1% [5.4%–6.7%]), although these differences appeared to be largely explained by the younger age of those with children in the household. Cigarette consumption also varied geographically, with the highest consumption in the North East of England (11.7 [10.6–12.8] cigarettes per day) and Scotland (11.7 [10.8–12.5]) and the lowest in London (8.4 [7.9–9.0]) and the South West (9.5 [8.9–10.1]).

**Conclusions:**

An estimated 28.6 billion cigarettes are smoked in Great Britain each year. There are persistent disparities in cigarette consumption across socioeconomic groups, regions, and nations, reflecting broader patterns of health inequality.

**Implications:**

While smoking prevalence has declined and regional differences have narrowed over recent decades, strong socioeconomic and geographic disparities in consumption remain, with particularly high levels among less advantaged groups and in regions with historically poorer health outcomes, such as the North East and Scotland. Addressing these disparities through targeted public health efforts and cessation support could contribute meaningfully to reducing health inequalities across Great Britain.

## Introduction

Cigarettes are the only legal product that, when used as intended, kill up to two thirds of those who use them.^1^ Smoking is also a major contributor to health inequalities.^2^^,^^3^ People who smoke more cigarettes per day are at greater risk of smoking-related harm^4^^-^^11^ and typically find it harder to quit.^12^^,^^13^ Children living in households with people who consume more cigarettes are exposed to more second-hand smoke^14^ and may be more likely to go on to take up smoking themselves.^15^^,^^16^ Consumption tends to be higher among people from less advantaged socioeconomic groups who smoke (e.g., those living in deprived areas or working in routine and manual occupations).^17^^,^^18^

In Great Britain, smoking prevalence has fallen substantially over recent decades.^19^ Over this period, there has also been a decline in the average number of cigarettes consumed by adults who smoke, from around 15 per day in the early 1990s to around 10 per day in 2022/2023.^18^^,^^20^ However, some continue to smoke heavily: in 2022, ~3% of adults in England reported smoking more than 20 cigarettes per day.^20^

Differences in smoking prevalence across nations and regions of Great Britain have been well characterized, and broadly reflect geographic inequalities in health and life expectancy. Health outcomes are generally poorer, and smoking rates have historically been higher, in Scotland and Wales compared with England and in regions in the north of England compared with the south,^19^^,^^21-27^ although disparities in smoking rates across regions in England have narrowed in recent years.^28^ Within each nation and region, smoking rates are also higher among people from less advantaged socioeconomic groups.^19^^,^^28^

The extent to which cigarette consumption varies across Great Britain, within nations and regions, is unclear. If there are areas where people tend to smoke more heavily (indicating higher levels of dependence^12^), more intensive support may be required to achieve the same reductions in smoking prevalence as in other areas where cigarette consumption is lower. There may also be implications for children’s exposure to second-hand smoke^14^ and for uptake of smoking, given that children are more likely to take up smoking if their parents or siblings smoke and this association is stronger for those with greater exposure to smoking by family members.^15^^,^^16^ Understanding variation in cigarette consumption, by geography and by socioeconomic position and the presence of children in the household, can inform public health strategies to reduce smoking, health inequalities, and intergenerational transmission of smoking. Smoking also has significant environmental consequences. Discarded cigarette butts are the most commonly littered item worldwide, containing plastics and toxic chemicals that can persist in ecosystems for years.^29^ Understanding patterns of cigarette consumption is therefore important not only for public health but also for environmental protection.

Using cross-sectional data from a nationally representative household survey, this study aimed to provide up-to-date estimates of cigarette consumption across Great Britain. We explored differences by socioeconomic position (indexed by occupational social grade), the presence of children in the household, and across nations and regions in (i) mean daily cigarette consumption (among adults who smoke cigarettes), (ii) the proportion of adults who smoke cigarettes consuming more than 20 cigarettes per day, and (iii) annual per-capita cigarette consumption (among adults). We also estimated the total number of cigarettes each nation and region consumes annually.

## Methods

### Pre-Registration

The study protocol and analysis plan were pre-registered on Open Science Framework (https://osf.io/f9842/).

### Design

Data were drawn from the Smoking Toolkit Study, an ongoing monthly cross-sectional survey of a representative sample of adults (≥16 years) in Great Britain.^30^^,^^31^ The study uses a hybrid of random probability and simple quota sampling to select a new sample of ~ 2450 adults each month. Data are collected through telephone interviews. Comparisons with other national surveys indicate the survey achieves nationally representative estimates of key variables such as sociodemographic characteristics and smoking prevalence.^30^ In addition, comparisons with sales data show the survey’s assessment of self-reported cigarette consumption produces reliable estimates.^32^

The present analyses used data collected between January 2022 and September 2024, aggregated across survey waves. We selected this period to provide up-to-date estimates while ensuring sufficient sample sizes within each region.

### Measures

Nations were categorized as England, Wales, and Scotland. Regions in England were categorized as North East, North West, Yorkshire and the Humber, East Midlands, West Midlands, East of England, London, South East, and South West.

Smoking status was assessed by asking participants which of the following best applied to them: (a) I smoke cigarettes (including hand-rolled) every day; (b) I smoke cigarettes (including hand-rolled), but not every day; (c) I do not smoke cigarettes at all, but I do smoke tobacco of some kind (e.g., pipe, cigar, or shisha); (d) I have stopped smoking completely in the last year; (e) I stopped smoking completely more than a year ago; (f) I have never been a smoker (i.e., smoked for a year or more). Responses a and b were considered current cigarette smoking.

Cigarette consumption was assessed among those who reported cigarette smoking with the question: “How many cigarettes do you usually smoke?”. Participants who responded “don’t know” were encouraged to give their best estimates. Participants could choose to give their answers per day or per week (or per month, in some waves). We calculated total daily cigarette consumption (i.e., cigarettes per day; or cigarettes per week divided by 7 or per month divided by 30). We considered consumption of more than 80 cigarettes per day to be implausible, so replaced responses above this level with the value 80 (*n* = 12).^18^ Consumption was imputed as 0 for participants who did not report current cigarette smoking.

Socioeconomic position was indexed using occupational social grade based on National Readership Survey classifications^33^ as AB (higher and intermediate managerial, administrative, or professional roles), C1 (supervisory, clerical, or junior managerial, administrative, or professional roles), C2 (skilled manual workers), D (semi-skilled and unskilled manual workers), and E (state pensioners, casual and lowest grade workers, unemployed with state benefits only). For analyses of differences in cigarette consumption by social grade, we collapsed these categories to ABC1 and C2DE. Children in the household were categorized as yes (≥1) or no (0). Age was categorized as 16–24, 25–34, 35–44, 45–54, 55–64, and 65 or above years. Gender was self-reported as man, woman, or in another way.

### Statistical Analysis

Data were analyzed in R. We excluded participants with missing data on smoking status or (for participants who reported cigarette smoking) cigarette consumption. Missing data on other variables were excluded on a per-analysis basis.

The Smoking Toolkit Study uses raking to weight the sample to match the populations in England, Wales, and Scotland. These profiles are determined each month by combining data from the UK Census, the Office for National Statistics mid-year estimates, and the annual National Readership Survey.^30^ The following analyses used data weighted to match the population of the respective nation. Sample sizes are reported unweighted.

We reported descriptive data on the sociodemographic profile of (a) adults and (b) adults who smoke cigarettes within each nation and region. We then estimated (i) the proportion of adults who smoke cigarettes, (ii) the mean daily cigarette consumption among adults who smoke cigarettes, (iii) the proportion of adults who smoke cigarettes consuming more than 20 cigarettes per day, and (iv) the annual mean per-capita cigarette consumption. Annual per-capita consumption was calculated as $( mean\ daily\ cigarette consumption\ among\ adults\ who\ smoke\ cigarettes\times proportion of\ adults\ who\ report\ smoking\ cigarette s)\times 365$. We used a Monte Carlo simulation to estimate 95% confidence intervals (CIs) for annual per-capita consumption.

We derived these estimates for the total population, overall and stratified by occupational social grade (ABC1/C2DE) and presence of children in the household (yes/no), and for each nation and region. We also reported estimates for (i), (ii), and (iv) within each region stratified by occupational social grade and presence of children in the household, but not for (iii) because the low prevalence of this outcome combined with relatively small sample sizes within each subgroup meant there was substantial imprecision in the estimates.

Using the latest mid-year estimates of population size,^34^ we also estimated the total number of cigarettes consumed per year within each nation and region (calculated as $annual\ per\ capita\ consumption\times population\ size$). We plotted the results by nation and region as heatmaps to display geographic variation in cigarette consumption.

In an unplanned analysis, we explored whether differences in mean daily cigarette consumption between adults who smoke cigarettes who did and did not have children in the household were explained by age differences between the two groups (given younger people who smoke tend to report consuming fewer cigarettes per day^18^). We used regression models to test the associations between children in the household and daily cigarette consumption (linear regression) and smoking more than 20 cigarettes per day (binary logistic regression), with and without adjustment for age.

## Results

A total of 78 978 adults aged 16 or above years in Great Britain were surveyed between January 2022 and September 2024 (mean [SD] = 2393 [97] per monthly wave), of whom 10 115 reported current cigarette smoking. We excluded 588 participants who did not report their smoking status and a further 594 participants who said they smoked cigarettes but did not report their cigarette consumption. This left a final analytic sample of 77 796 participants in Great Britain, of whom 9521 reported current cigarette smoking. Sample sizes and characteristics by nation and region are summarized in Table S1 (adults) and Table S2 (adults who smoked cigarettes).

### Cigarette Consumption in Great Britain

**Figure 1:**
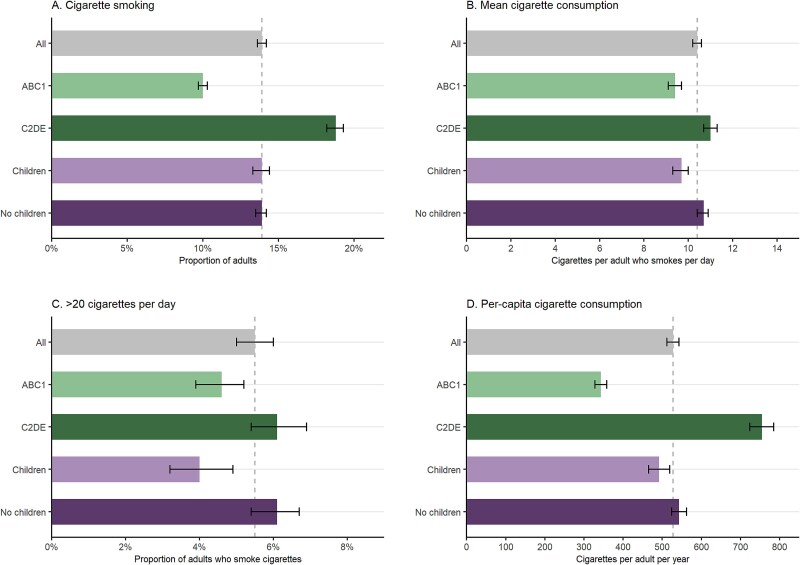
Cigarette consumption in Great Britain, 2022–2024, overall and by occupational social grade (ABC1 = more advantaged/C2DE = less advantaged) and the presence of children in the household (yes/no). Panels show (A) the proportion of adults who smoke, (B) the mean daily number of cigarettes consumed by adults who smoke cigarettes, (C) the proportion of adults who smoke cigarettes who smoke more than 20 cigarettes per day, and (D) the annual number of cigarettes consumed per-capita in Great Britain. Error bars represent 95% confidence intervals. Estimates with 95% CIs are provided in Table 1 (all), Table S4 (by occupational social grade), and Table S5 (by presence of children in the household).

Estimates of cigarette smoking prevalence and consumption in Great Britain are summarized in Table 1 and Figure 1.

**Table 1:** Cigarette Consumption in Great Britain, Overall and by Occupational Social Grade, Children in the Household, Nation, and Region

		**Consumption among adults who smoke cigarettes**		**Per-capita consumption**	**Total consumption**
	**Cigarette smoking** ^**1**^ **, % [95%CI]**	**Cigarettes per day, mean [95%CI]**	**>20 cigarettes per day, % [95%CI]**	**Cigarettes per year [95%CI]** ^**2**^	**Cigarettes per year (billions) [95%CI]** ^**3**^

Great Britain					
All adults	13.9 [13.6–14.2]	10.4 [10.2–10.6]	5.5 [5.0–6.0]	528 [512–543]	28.6 [27.8–29.5]
ABC1 (more advantaged)	10.0 [9.7–10.3]	9.4 [9.1–9.7]	4.6 [3.9–5.2]	343 [328–358]	–
C2DE (less advantaged)	18.8 [18.2–19.3]	11.0 [10.7–11.3]	6.1 [5.4–6.9]	755 [724–785]	–
Children in the household	13.9 [13.3–14.4]	9.7 [9.3–10.0]	4.0 [3.2–4.9]	492 [465–519]	–
No children in the household	13.9 [13.5–14.2]	10.7 [10.4–10.9]	6.1 [5.4–6.7]	543 [524–562]	–
Nation					
England	13.9 [13.6–14.3]	10.3 [10.1–10.5]	5.4 [4.8–6.0]	523 [506–539]	24.6 [23.8–25.4]
Wales	13.8 [12.7–15.0]	10.2 [9.6–10.9]	4.4 [2.9–5.9]	514 [462–568]	1.34 [1.21–1.49]
Scotland	13.3 [12.5–14.2]	11.7 [10.8–12.5]	7.3 [5.4–9.2]	568 [514–624]	2.61 [2.36–2.87]
Region in England					
North East	14.0 [12.4–15.5]	11.7 [10.6–12.8]	7.2 [4.1–10.4]	598 [512–688]	1.34 [1.15–1.54]
North West	13.9 [13.0–14.8]	10.9 [10.3–11.5]	6.7 [4.9–8.6]	553 [507–600]	3.42 [3.13–3.71]
Yorkshire and the Humber	13.3 [12.3–14.3]	11.0 [10.3–11.8]	5.1 [3.3–6.9]	534 [480–589]	2.44 [2.19–2.69]
East Midlands	14.5 [13.3–15.6]	10.8 [10.0–11.6]	7.2 [5.0–9.4]	572 [511–634]	2.34 [2.09–2.59]
West Midlands	13.4 [12.4–14.4]	10.5 [9.9–11.2]	5.3 [3.5–7.2]	514 [464–565]	2.52 [2.28–2.77]
East of England	14.1 [13.1–15.1]	11.1 [10.3–11.8]	6.2 [4.2–8.2]	571 [515–630]	3.00 [2.70–3.31]
London	13.8 [13.0–14.5]	8.4 [7.9–9.0]	3.8 [2.7–5.0]	423 [388–460]	3.07 [2.81–3.34]
South East	13.7 [12.9–14.6]	10.2 [9.7–10.8]	5.2 [3.8–6.5]	510 [470–553]	3.94 [3.63–4.27]
South West	15.0 [13.9–16.0]	9.5 [8.9–10.1]	3.8 [2.3–5.2]	520 [472–570]	2.52 [2.28–2.76]

^1^Proportion of adults who report currently smoking cigarettes (including hand-rolled) daily or non-daily.

^2^Mean number of cigarettes smoked per adult per year. Calculated as: (mean daily cigarette consumption among adults who smoke cigarettes × proportion of adults who smoke cigarettes) × 365. 95% CIs were derived using Monte Carlo simulations.

^3^Total number of cigarettes smoked by adults each year (in billions). Calculated as: annual per-capita consumption × adult population size. 95% CIs were derived by substituting annual per-capita consumption with the lower and upper 95% CI estimates for annual per-capita consumption.

Corresponding estimates within nations and regions stratified by social grade and the presence of children in the household are provided in Tables S3–S6.

Overall, the weighted prevalence of cigarette smoking was 13.9%. Participants who smoked cigarettes reported smoking on average 10.4 cigarettes per day, with 5.5% smoking more than 20 per day. Average per-capita consumption was 528 cigarettes per year, equating to a total consumption of 28.6 billion cigarettes in Great Britain per year.

Participants from less advantaged social grades were more likely to smoke cigarettes than those who were more advantaged (18.8% vs. 10.0%) and to smoke more heavily (mean consumption: 11.0 vs. 9.4 cigarettes per day; 6.1% vs. 4.6% smoking more than 20 per day). As a result, average per-capita consumption was more than twice as high among those from less compared with more advantaged social grades (755 vs. 343 cigarettes per year).

Cigarette smoking prevalence was similar between participants with (13.9%) and without (13.9%) children in the household. However, participants who smoked who had children in the household reported smoking less heavily than those who did not (mean consumption: 9.7 vs. 10.7 cigarettes per day; 4.0% vs. 6.1% smoking more than 20 per day). This difference appeared to be largely explained by those who smoked and had children in the household being younger on average than those without children in the household (Table S3); after adjusting for age, the difference in consumption between groups reduced from −1.01 (95%CI −1.45 to −0.56) to −0.29 (−0.77 to 0.19) cigarettes per day and the odds ratio for smoking more than 20 per day changed from 0.65 (95%CI: 0.51 to 0.83) to 0.75 (0.46 to 1.21). Average per-capita consumption was slightly lower among those with children in the household compared with those without (492 vs. 543 cigarettes per day).

### National and Regional Differences in Cigarette Consumption across Great Britain

**Figure 2:**
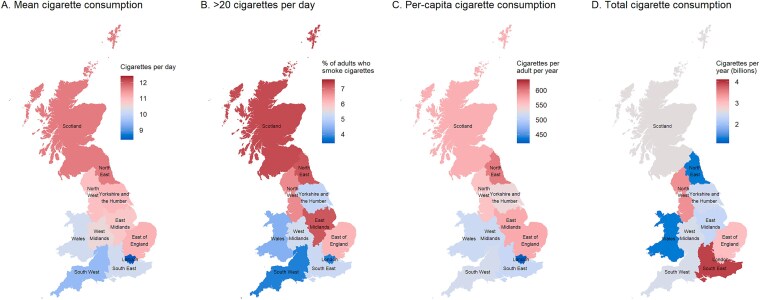
Cigarette consumption across Great Britain, 2022–2024. Panels show (A) the mean daily number of cigarettes consumed by adults who smoke cigarettes, (B) the proportion of adults who smoke cigarettes who smoke more than 20 cigarettes per day, (C) the annual number of cigarettes consumed per-capita, and (D) the total number of cigarettes consumed per year, within Scotland, Wales, and regions of England. Estimates are weighted to match the population of the respective nation. For each panel, the legend is centered on the national average for Great Britain (or the average across regions in England, Scotland, and Wales, for total cigarette consumption); therefore, gray shading indicates the estimate is similar to the national average, blue shading indicates the estimate is below average, and red shading indicates the estimate is above average. Estimates with 95% CIs are provided in Table 1. Corresponding figures stratified by social grade and the presence of children in the household are provided in Figures S1 and S2, respectively.

Estimates of cigarette consumption by nation and region within Great Britain are summarized in Table 1 and Figure 2. Corresponding estimates stratified by occupational grade and by the presence of children in the household are provided in Tables S4 and S5 and Figures S1 and S2.

Cigarette smoking prevalence was broadly similar across regions, ranging from 13.3% in Scotland and 13.4% in the West Midlands to 15.0% in the South West (95%CIs overlapped for all regional estimates). However, there was variation in cigarette consumption among those who smoked. The North East and Scotland had both the highest mean daily cigarette consumption (11.7 and 11.7 per day) and the highest proportions smoking more than 20 per day (7.3% and 7.2%), while London and the South West had the lowest (8.4 and 9.5 cigarettes per day; 3.8% and 3.8% smoking >20 per day). Average per-capita cigarette consumption was highest in the North East (598 per year) and lowest in London (423 per year). However, when we accounted for population size, the North East had the *lowest* total cigarette consumption (1.34 billion per year), along with Wales (1.34 billion per year). The largest number of cigarettes were consumed in the South East (3.94 billion per year), followed by the North West (3.42 billion per year).

There were some geographic differences in patterns of cigarette consumption by occupational social grade and the presence of children in the household. For example, average consumption was highest among less advantaged adults who smoked in Scotland (12.5 cigarettes per day) and lowest among more advantaged adults who smoked in London (7.4 cigarettes per day). Across all nations and regions, average per-capita cigarette consumption was above the national average for Great Britain among those from less advantaged social grades (highest in the East of England, 935 cigarettes per year) and below the national average among those who were more advantaged (lowest in London, 292 cigarettes per year). Average per-capita cigarette consumption was geographically more similar among those with and without children in the household.

## Discussion

This study provides a comprehensive update on cigarette consumption in Great Britain, with four key findings.

First, despite declines in smoking prevalence and mean cigarette consumption over recent decades,^18^^,^^19^ adults in Great Britain still consume an estimated 28.4 billion cigarettes each year. This has substantial public health, economic, and environmental implications. Smoking is a leading cause of preventable disease and death^1^ and it places a considerable burden on health and social care systems. In England alone, smoking is estimated to cost the NHS and social care sector £1.9 billion and £1.1 billion annually, respectively.^35^ With 24.6 billion cigarettes smoked in England each year, each cigarette equates to around £0.08 in healthcare costs and £0.04 in social care costs. In addition, cigarette butts are a significant source of litter and environmental waste.^29^ If all 28.6 billion cigarette butts in Great Britain are discarded, this could amount to around 140 000 metric tons of waste annually (assuming each butt weighs around 5 g). Many of these butts contain plastics that persist in the environment for years.^29^

Second, there are persistent socioeconomic disparities in cigarette smoking across Great Britain. Research has shown a strong link between smoking and socioeconomic position, with less advantaged groups smoking at higher rates, experiencing higher levels of dependence, and facing greater harm from smoking than their more advantaged counterparts.^17^ Our findings indicate that adults from less advantaged social grades who smoke consumed an average of 11.0 cigarettes per day, compared with 9.4 per day among those who were more advantaged. They were also more likely to smoke heavily, with 6.1% vs. 4.6% reporting that they smoked more than 20 cigarettes per day. Although these differences in daily consumption may seem modest, after accounting also for the higher smoking prevalence among less advantaged groups, the per-capita cigarette consumption in these groups is more than twice as high. Moreover, these differences may understate the degree of socioeconomic disparity in tobacco exposure. Even when adjusting for self-reported cigarette consumption, greater disadvantage is associated with higher levels of cotinine (an objective biochemical marker of tobacco use).^36^ This may be because people from less advantaged socioeconomic positions smoke each cigarette more intensely; studies show that people can modify the way they smoke each cigarette to achieve the desired nicotine dose, for example varying the number of puffs or how deeply they inhale—a behavior known as “titration.”^37^ Alternatively, there may be other explanations, such as higher use of illicit tobacco or other tobacco products (which may have higher nicotine content). Whatever the reason, it seems that less advantaged people are not only more likely to smoke and to smoke more heavily, they also experience greater toxicant exposure from each cigarette—all of which maintains health inequalities.

Third, while smoking prevalence was relatively similar across nations and regions, there were disparities in cigarette consumption. For example, the North East and Scotland had the highest mean daily cigarette consumption and the highest rates of heavy smoking, while London and the South West had the lowest. Socioeconomic disparities in cigarette consumption were also evident within each region. These geographic differences are in line with broader health inequalities across Great Britain, with poorer outcomes observed in Scotland and northern England compared with the south of England.^19^^,^^21-27^ Until recently, smoking prevalence followed the same pattern, but greater progress has been made in bringing down smoking rates in the northern regions of England in recent years than in the south, narrowing regional disparities in the proportion of adults who smoke.^28^ However, the present findings suggest the same pattern has not been seen for cigarette consumption among those who smoke. Addressing both regional and socioeconomic disparities in smoking—especially targeting areas with higher per-capita consumption and targeting less advantaged groups—could play an important role in reducing health inequalities across Great Britain.

Finally, cigarette consumption was slightly lower among adults who smoked who had children in the household. Those with children averaged one fewer cigarette per day than those without (9.7 vs. 10.7 per day) and were less likely to report heavy smoking (4.0% vs. 6.1% smoked >20 cigarettes per day). However, when we adjusted for age differences between those with and without children in the household, the difference in mean daily consumption was attenuated. This suggests that difference in consumption may be more related to the younger average age of people who have children than to an independent effect of having children in the household. Recent evidence (also from the Smoking Toolkit Study) shows younger age is associated with smoking fewer cigarettes per day.^18^ In addition to differences in age, previous research indicates that people with children—especially young children—are more likely to adopt measures to reduce second-hand smoke exposure, such as restricting smoking in the home and car.^38-40^ Such restrictions might contribute to lower consumption by limiting opportunities to smoke.

While these findings are specific to Great Britain, they have broader implications for global tobacco control. Socioeconomic and geographic disparities in smoking behavior are seen worldwide, particularly in high-income countries.^2^^,^^17^ Our results underscore the importance of not only reducing smoking prevalence but also addressing disparities in cigarette consumption and intensity of use—factors that contribute to maintaining health inequalities. Tailored tobacco control policies that account for regional and socioeconomic variation, such as targeted cessation support, may be necessary globally to reduce the overall burden of smoking and associated inequalities.

This study had several limitations. Although the overall sample size was large, subgroup sample sizes were relatively small within specific regions. The survey did not assess where people smoked, so we could not explore whether people with children in the household were less likely to smoke in the home or while around their children. In addition, as a household survey, the sample excluded people living in institutions or experiencing homelessness. As these groups have higher smoking rates,^41^^,^^42^ our estimates likely underestimate overall cigarette consumption. Future studies could address this by incorporating data from targeted surveys to improve representativeness.

In conclusion, an estimated 28.6 billion cigarettes were smoked in Great Britain each year between 2022 and 2024. There were persistent disparities in cigarette consumption across socioeconomic groups, regions, and nations, reflecting broader patterns of health inequality. While smoking prevalence has declined and regional differences have narrowed over recent decades,^19^^,^^28^ strong socioeconomic and geographic disparities in consumption remain, with particularly high levels among less advantaged groups and in regions with historically poorer health outcomes, such as the North East and Scotland. Addressing these disparities through targeted public health efforts and cessation support could contribute meaningfully to reducing health inequalities across Great Britain.

## Supplementary Material

Supplementary_file_ntaf133

## Data Availability

Data are available on Open Science Framework (https://osf.io/f9842/).

## References

[1] Reitsma MB, Fullman N, Ng M, et al. Smoking prevalence and attributable disease burden in 195 countries and territories, 1990–2015: a systematic analysis from the global burden of disease study 2015. Lancet. 2017;389(10082):1885-1906. 10.1016/S0140-6736(17)30819-X 28390697 PMC5439023

[2] Marmot M . Smoking and inequalities. Lancet. 2006;368(9533):341-342. 10.1016/S0140-6736(06)68976-9 16876643

[3] Jha P, Ramasundarahettige C, Landsman V, et al. 21st-century hazards of smoking and benefits of cessation in the United States. N Engl J Med. 2013;368(4):341-350. 10.1056/NEJMsa1211128 23343063

[4] Hackshaw A, Morris JK, Boniface S, Tang JL, Milenković D. Low cigarette consumption and risk of coronary heart disease and stroke: meta-analysis of 141 cohort studies in 55 study reports. BMJ. 2018;360:j5855. 10.1136/bmj.j5855 29367388 PMC5781309

[5] Bjartveit K, Tverdal A. Health consequences of smoking 1–4 cigarettes per day. Tob Control. 2005;14(5):315-320. 10.1136/tc.2005.011932 16183982 PMC1748107

[6] Doll R, Peto R, Boreham J, Sutherland I. Mortality in relation to smoking: 50 years’ observations on male British doctors. BMJ. 2004;328(7455):1519. 10.1136/bmj.38142.554479.AE 15213107 PMC437139

[7] Shah RS, Cole JW. Smoking and stroke: the more you smoke the more you stroke. Expert Rev Cardiovasc Ther. 2010;8(7):917-932. 10.1586/erc.10.56 20602553 PMC2928253

[8] Brennan P, Bogillot O, Cordier S, et al. Cigarette smoking and bladder cancer in men: a pooled analysis of 11 case-control studies. Int J Cancer. 2000;86(2):289-294. 10.1002/(SICI)1097-0215(20000415)86:2<289::AID-IJC21>3.0.CO;2-M 10738259

[9] Manson JE, Ajani UA, Liu S, Nathan DM, Hennekens CH. A prospective study of cigarette smoking and the incidence of diabetes mellitus among us male physicians. Am J Med. 2000;109(7):538-542. 10.1016/S0002-9343(00)00568-4 11063954

[10] Rimm EB, Manson JE, Stampfer MJ, et al. Cigarette smoking and the risk of diabetes in women. Am J Public Health. 1993;83(2):211-214. 10.2105/AJPH.83.2.211 8427325 PMC1694562

[11] Pirie K, Peto R, Reeves GK, Green J, Beral V, Million Women Study Collaborators. The 21st century hazards of smoking and benefits of stopping: a prospective study of one million women in the UK. Lancet. 2013;381(9861):133-141. 10.1016/S0140-6736(12)61720-6 23107252 PMC3547248

[12] Fagerström K . Time to first cigarette; the best single indicator of tobacco dependence? Monaldi Arch Chest Dis. 2003;59(1):91-94 14533289

[13] Zhou X, Nonnemaker J, Sherrill B, Gilsenan AW, Coste F, West R. Attempts to quit smoking and relapse: factors associated with success or failure from the ATTEMPT cohort study. Addict Behav. 2009;34(4):365-373. 10.1016/j.addbeh.2008.11.013 19097706

[14] Wipfli H, Avila-Tang E, Navas-Acien A, et al. Secondhand smoke exposure among women and children: evidence from 31 countries. Am J Public Health. 2008;98(4):672-679. 10.2105/AJPH.2007.126631 18309121 PMC2376995

[15] Leonardi-Bee J, Jere ML, Britton J. Exposure to parental and sibling smoking and the risk of smoking uptake in childhood and adolescence: a systematic review and meta-analysis. Thorax. 2011;66(10):847-855. 10.1136/thx.2010.153379 21325144

[16] Gilman SE, Rende R, Boergers J, et al. Parental smoking and adolescent smoking initiation: an intergenerational perspective on tobacco control. Pediatrics. 2009;123(2):e274-e281. 10.1542/peds.2008-2251 19171580 PMC2632764

[17] Hiscock R, Bauld L, Amos A, Fidler JA, Munafò M. Socioeconomic status and smoking: a review. Ann N Y Acad Sci. 2012;1248(1):107-123. 10.1111/j.1749-6632.2011.06202.x 22092035

[18] Jackson SE, Tattan-Birch H, Buss V, Shahab L, Brown J. Trends in daily cigarette consumption among smokers: a population study in England, 2008–2023. Nicotine Tob Res. 2025;27(4):722-732. 10.1093/ntr/ntae071 38692652 PMC11997653

[19] Office for National Statistics . Adult Smoking Habits in the UK: 2023; 2024. Accessed October 1, 2024. https://www.ons.gov.uk/peoplepopulationandcommunity/healthandsocialcare/healthandlifeexpectancies/bulletins/adultsmokinghabitsingreatbritain/2023.

[20] NHS Digital . Health Survey for England, 2022 Part 1. NHS Digital; 2024. Accessed October 25, 2024. https://digital.nhs.uk/data-and-information/publications/statistical/health-survey-for-england/2022-part-1/adults-health-related-behaviours.

[21] Beard E, Brown J, West R, Angus C, Kaner E, Michie S. Healthier Central England or North–South divide? Analysis of national survey data on smoking and high-risk drinking. BMJ Open. 2017;7(3):e014210. 10.1136/bmjopen-2016-014210 PMC5353327 28249851

[22] Ellis A, Robert F. Regional health inequalities in England. Reg Trends. 2010;42(1):60-79. 10.1057/rt.2010.5

[23] Office for National Statistics . Life expectancy for local areas in England, Northern Ireland and Wales; 2024. Accessed August 13, 2024. https://www.ons.gov.uk/peoplepopulationandcommunity/healthandsocialcare/healthandlifeexpectancies/bulletins/lifeexpectancyforlocalareasoftheuk/between2001to2003and2020to2022#life-expectancy-for-local-areas-in-england-northern-ireland-and-wales-between-2001-to-2003-and-2020-to-2022-data.

[24] Bennett JE, Li G, Foreman K, et al. The future of life expectancy and life expectancy inequalities in England and Wales: Bayesian spatiotemporal forecasting. Lancet. 2015;386(9989):163-170. 10.1016/S0140-6736(15)60296-3 25935825 PMC4502253

[25] Seaman R, Leyland AH, Popham F. Increasing inequality in age of death at shared levels of life expectancy: a comparative study of Scotland and England and Wales. SSM Popul Health. 2016;2:724-731. 10.1016/j.ssmph.2016.10.001 28018961 PMC5165049

[26] Woods LM, Rachet B, Riga M, Stone N, Shah A, Coleman MP. Geographical variation in life expectancy at birth in England and Wales is largely explained by deprivation. J Epidemiol Community Health. 2005;59(2):115-120. 10.1136/jech.2003.013003 15650142 PMC1733001

[27] Leyland AH . Increasing inequalities in premature mortality in Great Britain. J Epidemiol Community Health. 2004;58(4):296-302. 10.1136/jech.2003.007278 15026442 PMC1732731

[28] Jackson SE, Cox S, Buss V, Tattan-Birch H, Brown J. Trends in smoking prevalence and socioeconomic inequalities across regions in England: a population study, 2006 to 2024. Published online October 24, 2024. 10.1101/2024.10.24.24316046 PMC12319661 40101759

[29] Vanapalli KR, Sharma HB, Anand S, et al. Cigarettes butt littering: the story of the world’s most littered item from the perspective of pollution, remedial actions, and policy measures. J Hazard Mater. 2023;453:131387. 10.1016/j.jhazmat.2023.131387 37080035

[30] Fidler JA, Shahab L, West O, et al. “The smoking toolkit study”: a national study of smoking and smoking cessation in England. BMC Public Health. 2011;11(1):479. 10.1186/1471-2458-11-479 21682915 PMC3145589

[31] Kock L, Shahab L, Moore G, et al. Protocol for expansion of an existing national monthly survey of smoking behaviour and alcohol use in England to Scotland and Wales: the smoking and alcohol toolkit study. Wellcome Open Res. 2021;6:67. 10.12688/wellcomeopenres.16700.1 34458587 PMC8370132

[32] Jackson SE, Beard E, Kujawski B, et al. Comparison of trends in self-reported cigarette consumption and sales in England, 2011 to 2018. JAMA Netw Open. 2019;2(8):e1910161. 10.1001/jamanetworkopen.2019.10161 31461148 PMC6716287

[33] National Readership Survey . Social grade—definitions and discriminatory power. Published online 2007. Accessed October 1, 2012. http://www.nrs.co.uk/lifestyle.html

[34] Office for National Statistics . Population estimates for the UK, England, Wales, Scotland and Northern Ireland: mid-2023; 2024. Accessed October 25, 2024. https://www.ons.gov.uk/peoplepopulationandcommunity/populationandmigration/populationestimates/bulletins/annualmidyearpopulationestimates/mid2023.

[35] Department of Health and Social Care . Stopping the Start: Our New Plan to Create a Smokefree Generation; 2023. Accessed October 5, 2023. https://www.gov.uk/government/publications/stopping-the-start-our-new-plan-to-create-a-smokefree-generation.

[36] Fidler JA, Jarvis MJ, Mindell J, West R. Nicotine intake in cigarette smokers in England: distribution and demographic correlates. Cancer Epidemiol Biomarkers. 2008;17(12):3331-3336. 10.1158/1055-9965.EPI-08-0296 19064547

[37] Ashton H, Stepney R, Thompson JW. Self-titration by cigarette smokers. Br Med J. 1979;2(6186):357-360. 486932 10.1136/bmj.2.6186.357 PMC1596095

[38] Hawkins SS, Berkman L. Parental home smoking policies: the protective effect of having a young child in the household. Prev Med. 2011;53(1–2):61-63. 10.1016/j.ypmed.2011.05.016 21679724

[39] Norman GJ, Ribisl KM, Howard-Pitney B, Howard KA. Smoking bans in the home and car: do those who really need them have them? Prev Med. 1999;29(6):581-589. 10.1006/pmed.1999.0574 10600441

[40] Borland R, Yong HH, Cummings KM, Hyland A, Anderson S, Fong GT. Determinants and consequences of smoke-free homes: findings from the International Tobacco Control (ITC) Four Country Survey. Tob Control. 2006;15(suppl 3):iii42-iii50. 10.1136/tc.2005.012492 16754946 PMC2593064

[41] Soar K, Dawkins L, Robson D, Cox S. Smoking amongst adults experiencing homelessness: a systematic review of prevalence rates, interventions and the barriers and facilitators to quitting and staying quit. J Smok Cessat. 2020;15(2):94-108. 10.1017/jsc.2020.11

[42] Kalman D, Morissette SB, George TP. Co-morbidity of smoking in patients with psychiatric and substance use disorders. Am J Addict. 2005;14(2):106-123. 10.1080/10550490590924728 16019961 PMC1199553

