# Supplementary material for: Sociodemographic and Regional Differences in Cigarette Consumption Across Great Britain: A Population Study, 2022–2024
Source: Nicotine Tob Res. 2025 Jul 18;27(12):2289–96. doi: 10.1093/ntr/ntaf133 (PMC12641182; doi:10.1093/ntr/ntaf133)
Supplement: Supplementary_file_ntaf133 [file supplementary_file_ntaf133.docx]

##### **Table S1.** Sample characteristics by nation and region: **adults**

|  | **Great Britain** | **Nation** | | |  | **Region in England** | | | | | | | | |
| --- | --- | --- | --- | --- | --- | --- | --- | --- | --- | --- | --- | --- | --- | --- |
|  |  | **England** | **Wales** | **Scotland** |  | **North East** | **North West** | **Yorkshire and the Humber** | **East Midlands** | **West Midlands** | **East of England** | **London** | **South East** | **South West** |
|  |  |  |  |  |  |  |  |  |  |  |  |  |  |  |
| **N** | 77,796 | 54,624 | 8,278 | 14,894 |  | 2,485 | 6,967 | 5,215 | 4,767 | 5,449 | 6,179 | 9,107 | 9,002 | 5,453 |
|  |  |  |  |  |  |  |  |  |  |  |  |  |  |  |
| **Age (years)** |  |  |  |  |  |  |  |  |  |  |  |  |  |  |
| 16-24 | 13.2 | 13.3 | 12.5 | 12.7 |  | 10.8 | 13.0 | 13.5 | 13.1 | 14.2 | 12.6 | 16.0 | 12.2 | 11.8 |
| 25-34 | 16.6 | 16.7 | 15.7 | 16.4 |  | 13.7 | 16.5 | 15.8 | 16.1 | 17.6 | 16.3 | 21.4 | 15.1 | 14.7 |
| 35-44 | 15.6 | 15.8 | 14.1 | 14.8 |  | 13.8 | 15.2 | 15.4 | 15.4 | 14.5 | 15.9 | 19.0 | 15.8 | 14.7 |
| 45-54 | 16.3 | 16.3 | 16.2 | 16.1 |  | 16.4 | 16.2 | 16.4 | 15.3 | 15.3 | 15.9 | 16.9 | 17.0 | 16.4 |
| 55-64 | 15.4 | 15.2 | 16.6 | 16.5 |  | 18.3 | 15.5 | 15.5 | 15.9 | 15.1 | 16.1 | 12.0 | 16.1 | 15.5 |
| 65+ | 22.9 | 22.7 | 24.9 | 23.4 |  | 27.0 | 23.5 | 23.4 | 24.2 | 23.3 | 23.3 | 14.7 | 23.8 | 26.9 |
| Missing, *n* | 17 | 17 | 12 | 15 |  | 0 | 3 | 2 | 1 | 4 | 2 | 1 | 2 | 2 |
|  |  |  |  |  |  |  |  |  |  |  |  |  |  |  |
| **Gender** |  |  |  |  |  |  |  |  |  |  |  |  |  |  |
| Men | 48.6 | 48.6 | 48.0 | 47.9 |  | 48.3 | 48.5 | 48.6 | 48.5 | 48.6 | 48.6 | 49.1 | 48.5 | 48.2 |
| Women | 50.7 | 50.5 | 51.7 | 51.6 |  | 51.0 | 50.6 | 50.6 | 50.5 | 50.5 | 50.7 | 49.8 | 50.6 | 50.8 |
| Other | 0.8 | 0.9 | 0.4 | 0.5 |  | 0.7 | 0.9 | 0.8 | 1.0 | 0.9 | 0.6 | 1.1 | 0.9 | 1.0 |
| Missing, *n* | 304 | 304 | 32 | 58 |  | 7 | 33 | 37 | 22 | 29 | 39 | 64 | 42 | 31 |
|  |  |  |  |  |  |  |  |  |  |  |  |  |  |  |
| **Social grade** |  |  |  |  |  |  |  |  |  |  |  |  |  |  |
| AB (most advantaged) | 26.4 | 26.7 | 23.2 | 24.4 |  | 25.8 | 25.5 | 24.1 | 23.6 | 24.7 | 26.9 | 30.7 | 28.9 | 25.9 |
| C1 | 29.8 | 29.7 | 30.7 | 29.9 |  | 27.9 | 29.2 | 28.0 | 29.2 | 28.2 | 30.7 | 33.7 | 29.8 | 27.4 |
| C2 | 20.3 | 20.3 | 19.5 | 20.2 |  | 20.1 | 20.5 | 21.9 | 22.8 | 21.1 | 21.0 | 16.3 | 19.6 | 22.7 |
| D | 14.4 | 14.3 | 15.6 | 15.7 |  | 14.8 | 14.8 | 15.9 | 15.3 | 16.2 | 13.1 | 11.8 | 13.6 | 14.9 |
| E (least advantaged) | 9.2 | 9.0 | 11.0 | 9.7 |  | 11.5 | 10.1 | 10.1 | 9.0 | 9.8 | 8.4 | 7.5 | 8.1 | 9.2 |
|  |  |  |  |  |  |  |  |  |  |  |  |  |  |  |
| **Children in the household** | 29.0 | 29.4 | 25.8 | 26.5 |  | 24.6 | 28.3 | 29.8 | 28.0 | 30.2 | 29.8 | 32.1 | 30.8 | 26.4 |
|  |  |  |  |  |  |  |  |  |  |  |  |  |  |  |

Data shown are percentages weighted to match the population of the respective nation, unless otherwise specified. Sample sizes and numbers of missing cases are presented as unweighted *n*s. There were no missing data on social grade or children in the household.

##### **Table S2.** Sample characteristics by nation and region: **adults who smoke cigarettes**

|  | **Great Britain** | **Nation** | | |  | **Region in England** | | | | | | | | |
| --- | --- | --- | --- | --- | --- | --- | --- | --- | --- | --- | --- | --- | --- | --- |
|  |  | **England** | **Wales** | **Scotland** |  | **North East** | **North West** | **Yorkshire and the Humber** | **East Midlands** | **West Midlands** | **East of England** | **London** | **South East** | **South West** |
|  |  |  |  |  |  |  |  |  |  |  |  |  |  |  |
| **N** | 9,521 | 7,016 | 867 | 1,638 |  | 325 | 914 | 654 | 625 | 692 | 774 | 1,176 | 1,103 | 753 |
|  |  |  |  |  |  |  |  |  |  |  |  |  |  |  |
| **Age (years)** |  |  |  |  |  |  |  |  |  |  |  |  |  |  |
| 16-24 | 16.3 | 16.3 | 18.3 | 15.4 |  | 16.0 | 15.2 | 15.5 | 14.2 | 15.3 | 15.8 | 17.1 | 18.6 | 17.2 |
| 25-34 | 23.1 | 23.2 | 17.6 | 23.1 |  | 18.4 | 24.0 | 23.0 | 20.7 | 23.3 | 21.6 | 28.2 | 24.0 | 20.0 |
| 35-44 | 17.4 | 17.4 | 19.2 | 18.0 |  | 15.6 | 15.5 | 18.9 | 19.0 | 14.7 | 19.1 | 16.6 | 17.3 | 19.5 |
| 45-54 | 16.9 | 17.0 | 18.4 | 16.3 |  | 19.3 | 16.7 | 17.6 | 18.0 | 16.8 | 18.1 | 16.9 | 15.4 | 16.4 |
| 55-64 | 13.6 | 13.5 | 13.9 | 13.5 |  | 15.1 | 13.9 | 12.8 | 15.7 | 15.3 | 13.5 | 11.9 | 13.0 | 13.0 |
| 65+ | 12.6 | 12.6 | 12.6 | 13.6 |  | 15.6 | 14.7 | 12.2 | 12.4 | 14.6 | 11.9 | 9.2 | 11.8 | 13.9 |
| Missing, *n* | 2 | 2 | 0 | 1 |  | 0 | 0 | 0 | 0 | 2 | 0 | 0 | 0 | 0 |
|  |  |  |  |  |  |  |  |  |  |  |  |  |  |  |
| **Gender** |  |  |  |  |  |  |  |  |  |  |  |  |  |  |
| Men | 52.5 | 52.6 | 49.5 | 51.6 |  | 49.8 | 51.1 | 52.7 | 50.6 | 53.7 | 54.9 | 56.7 | 50.8 | 50.2 |
| Women | 46.3 | 46.1 | 50.0 | 47.5 |  | 48.5 | 47.5 | 46.2 | 47.4 | 44.5 | 44.4 | 41.6 | 48.1 | 48.5 |
| Other | 1.2 | 1.4 | 0.4 | 0.9 |  | 1.6 | 1.4 | 1.1 | 1.9 | 1.8 | 0.7 | 1.7 | 1.1 | 1.3 |
| Missing, *n* | 41 | 41 | 3 | 4 |  | 0 | 5 | 6 | 2 | 5 | 5 | 7 | 7 | 4 |
|  |  |  |  |  |  |  |  |  |  |  |  |  |  |  |
| **Social grade** |  |  |  |  |  |  |  |  |  |  |  |  |  |  |
| AB (most advantaged) | 15.1 | 15.7 | 12.2 | 11.9 |  | 9.9 | 14.8 | 14.0 | 14.1 | 15.5 | 15.9 | 20.9 | 16.1 | 13.5 |
| C1 | 25.4 | 25.4 | 24.8 | 25.5 |  | 22.7 | 26.8 | 26.6 | 22.8 | 25.8 | 22.4 | 29.5 | 25.3 | 23.4 |
| C2 | 25.0 | 25.0 | 23.4 | 23.5 |  | 25.6 | 21.9 | 23.6 | 28.2 | 22.8 | 27.1 | 21.7 | 27.1 | 28.4 |
| D | 19.9 | 19.7 | 22.7 | 19.7 |  | 19.6 | 20.4 | 20.1 | 21.1 | 20.8 | 21.2 | 14.6 | 20.9 | 20.6 |
| E (least advantaged) | 14.5 | 14.2 | 16.9 | 19.3 |  | 22.2 | 16.1 | 15.7 | 13.7 | 15.1 | 13.4 | 13.3 | 10.7 | 14.1 |
|  |  |  |  |  |  |  |  |  |  |  |  |  |  |  |
| **Children in the household** | 29.0 | 29.5 | 27.8 | 24.0 |  | 23.4 | 26.5 | 34.6 | 31.8 | 30.0 | 30.9 | 25.0 | 32.2 | 29.8 |
|  |  |  |  |  |  |  |  |  |  |  |  |  |  |  |

Data shown are percentages weighted to match the population of the respective nation, unless otherwise specified. Sample sizes and numbers of missing cases are presented as unweighted *n*s. There were no missing data on social grade or children in the household.

##### **Table S3.** Age distribution of adults who smoke cigarettes who do and do not have children in the household

|  | **% [95% CI]** | |
| --- | --- | --- |
|  | **Children** | **No children** |
|  |  |  |
| Age (years) |  |  |
| 16-24 | 17.7 [16.0–19.5] | 15.7 [14.7–16.7] |
| 25-34 | 28.1 [26.0–30.2] | 21.1 [19.9–22.3] |
| 35-44 | 31.0 [29.0–33.1] | 11.8 [10.9–12.7] |
| 45-54 | 17.4 [15.8–19.0] | 16.7 [15.7–17.8] |
| 55-64 | 4.4 [3.6–5.2] | 17.4 [16.4–18.4] |
| ≥65 | 1.3 [0.9–1.8] | 17.3 [16.3–18.2] |
|  |  |  |

There were some missing data on age (see Tables S1 and S2); data shown are valid column percentages.


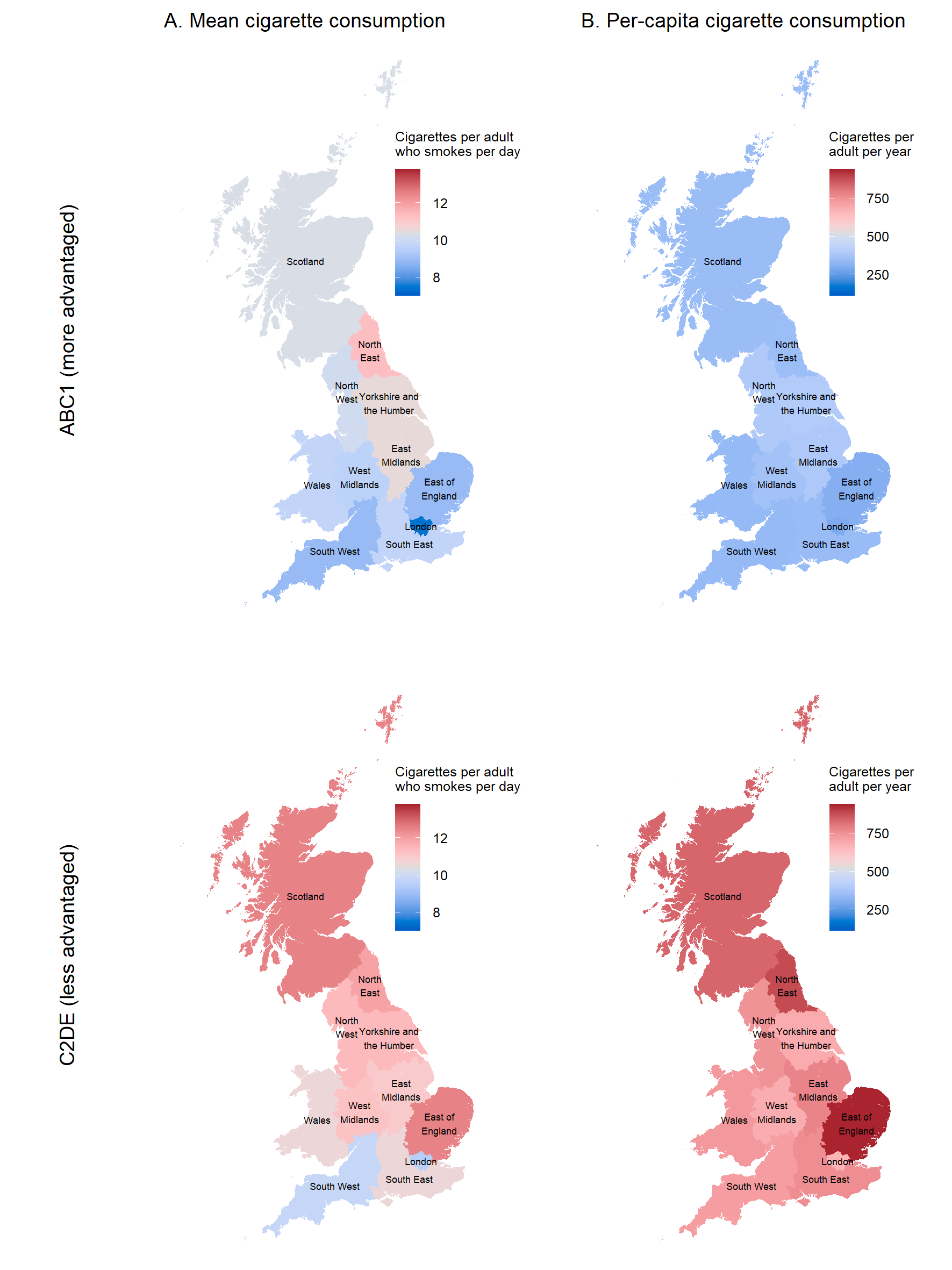


##### **Figure S1. Cigarette consumption by occupational social grade in Great Britain, 2022-24.** Panels show (A) the mean daily number of cigarettes consumed by adults who smoke cigarettes and (B) the annual number of cigarettes consumed per-capita, among more (ABC1) and less (C2DE) advantaged social grades within Scotland, Wales, and regions of England. Estimates are weighted to match the population of the respective nation. For each panel, grey shading indicates the estimate is similar to the national average for the respective social grade category in Great Britain, blue shading indicates the estimate is below average, and red shading indicates the estimate is above average. The same upper and lower limits were applied to the colour palettes across **Figure S1** and **Figure S2** to enable comparisons of subgroup differences; note these limits differ from those used in **Figure 2**. Estimates with 95% CIs are provided in **Table S3**.

##### **Table S4.** Cigarette consumption in Great Britain, by nation and region and occupational social grade

|  | **Cigarette smoking^1^, % [95%CI]** | |  | **Consumption among adults who smoke cigarettes** | | |  | | **Per-capita consumption** | |
| --- | --- | --- | --- | --- | --- | --- | --- | --- | --- | --- |
|  |  |  |  | **Cigarettes per day, mean [95%CI]** | | |  | | **Cigarettes per year [95%CI]^2^** | |
|  | **ABC1^3^** | **C2DE^3^** |  | **ABC1^3^** | **C2DE^3^** |  | | **ABC1^3^** | | **C2DE^3^** |
|  |  |  |  |  |  |  | |  | |  |
| Nation |  |  |  |  |  |  | |  | |  |
| England | 10.1 [9.8–10.5] | 18.8 [18.2–19.4] |  | 9.3 [9.0–9.6] | 10.9 [10.6–11.3] |  | | 343 [327–359] | | 748 [714–783] |
| Wales | 9.5 [8.4–10.6] | 18.9 [16.8–21.0] |  | 9.7 [8.8–10.6] | 10.6 [9.6–11.5] |  | | 336 [288–387] | | 731 [630–837] |
| Scotland | 9.2 [8.4–10.0] | 18.3 [16.8–19.8] |  | 10.3 [9.5–11.1] | 12.5 [11.3–13.8] |  | | 346 [306–387] | | 835 [729–946] |
|  |  |  |  |  |  |  | |  | |  |
| Region in England |  |  |  |  |  |  | |  | |  |
| North East | 8.5 [7.1–9.9] | 20.3 [17.4–23.2] |  | 11.3 [9.6–13.1] | 11.9 [10.5–13.3] |  | | 351 [274–434] | | 882 [720–1051] |
| North West | 10.6 [9.7–11.5] | 17.9 [16.3–19.6] |  | 10.1 [9.2–11.0] | 11.4 [10.6–12.3] |  | | 391 [344–439] | | 745 [659–834] |
| Yorkshire and the Humber | 10.4 [9.3–11.4] | 16.5 [14.7–18.3] |  | 10.5 [9.4–11.6] | 11.4 [10.3–12.5] |  | | 399 [341–458] | | 687 [588–788] |
| East Midlands | 10.1 [9.0–11.2] | 19.3 [17.3–21.4] |  | 10.5 [9.2–11.8] | 11.0 [10.0–12.0] |  | | 387 [325–453] | | 775 [670–886] |
| West Midlands | 10.4 [9.4–11.5] | 16.7 [14.9–18.4] |  | 9.6 [8.8–10.4] | 11.2 [10.2–12.1] |  | | 364 [318–414] | | 683 [592–778] |
| East of England | 9.4 [8.5–10.3] | 20.5 [18.6–22.4] |  | 8.9 [8.0–9.7] | 12.5 [11.4–13.5] |  | | 305 [264–349] | | 935 [819–1059] |
| London | 10.8 [10.0–11.5] | 19.2 [17.5–20.9] |  | 7.4 [6.8–7.9] | 9.5 [8.6–10.4] |  | | 292 [263–323] | | 666 [582–756] |
| South East | 9.7 [8.9–10.5] | 19.5 [17.9–21.1] |  | 9.7 [8.9–10.6] | 10.6 [9.8–11.4] |  | | 343 [304–386] | | 754 [674–841] |
| South West | 10.4 [9.3–11.4] | 20.2 [18.3–22.1] |  | 8.9 [8.1–9.7] | 9.8 [9.0–10.7] |  | | 338 [293–384] | | 723 [632–816] |
|  |  |  |  |  |  |  | |  | |  |

^1^ Proportion of adults who report currently smoking cigarettes (including hand-rolled) daily or non-daily.

^2^ Mean number of cigarettes smoked per adult per year. Calculated as: (mean daily cigarette consumption among adults who smoke cigarettes x proportion of adults who smoke cigarettes) x 365. 95% CIs were derived using Monte Carlo simulations.

^3^ ABC1 = more advantaged, C2DE = less advantaged.


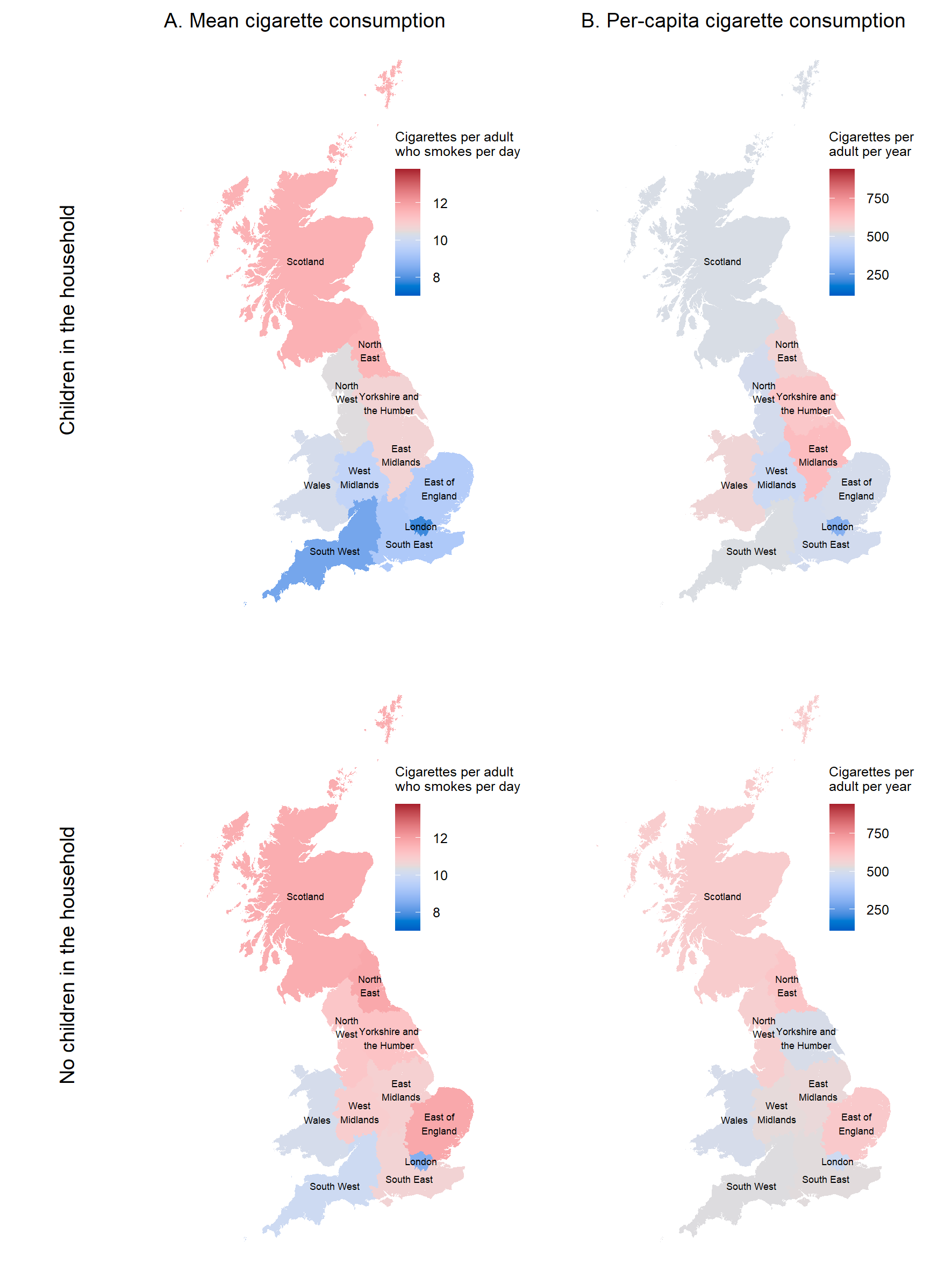


##### **Figure S2. Cigarette consumption by the presence of children in the household in Great Britain, 2022-24.** Panels show (A) the mean daily number of cigarettes consumed by adults who smoke cigarettes and (B) the annual number of cigarettes consumed per-capita, among those with and without children in the household within Scotland, Wales, and regions of England. Estimates are weighted to match the population of the respective nation. For each panel, grey shading indicates the estimate is similar to the national average for the respective children category in Great Britain, blue shading indicates the estimate is below average, and red shading indicates the estimate is above average. The same upper and lower limits were applied to the colour palettes across **Figure S1** and **Figure S2** to enable comparisons of subgroup differences; note these limits differ from those used in **Figure 2**. Estimates with 95% CIs are provided in **Table S4**.

##### **Table S5.** Cigarette consumption in Great Britain, by nation and region and children in the household

|  | **Cigarette smoking^1^, % [95%CI]** | |  | **Consumption among adults who smoke cigarettes** | | |  | | **Per-capita consumption** | |
| --- | --- | --- | --- | --- | --- | --- | --- | --- | --- | --- |
|  |  |  |  | **Cigarettes per day, mean [95%CI]** | | |  | | **Cigarettes per year [95%CI]^2^** | |
|  | **Children** | **No children** |  | **Children** | **No children** |  | | **Children** | | **No children** |
|  |  |  |  |  |  |  | |  | |  |
| Nation |  |  |  |  |  |  | |  | |  |
| England | 13.9 [13.3–14.6] | 13.9 [13.5–14.3] |  | 9.6 [9.2–10.0] | 10.6 [10.3–10.9] |  | | 487 [457–518] | | 538 [516–560] |
| Wales | 14.9 [12.3–17.4] | 13.5 [12.2–14.7] |  | 10.2 [8.9–11.6] | 10.2 [9.4–11.0] |  | | 555 [441–678] | | 503 [443–565] |
| Scotland | 12.1 [10.4–13.9] | 13.8 [12.8–14.7] |  | 11.6 [10.4–12.8] | 11.7 [10.7–12.8] |  | | 512 [422–606] | | 589 [524–658] |
|  |  |  |  |  |  |  | |  | |  |
| Region in England |  |  |  |  |  |  | |  | |  |
| North East | 13.3 [10.2–16.3] | 14.2 [12.4–16.0] |  | 11.5 [9.2–13.8] | 11.8 [10.5–13.1] |  | | 558 [394–739] | | 612 [510–718] |
| North West | 13.1 [11.4–14.8] | 14.3 [13.2–15.3] |  | 10.4 [9.1–11.6] | 11.1 [10.3–11.8] |  | | 497 [412–588] | | 579 [523–638] |
| Yorkshire and the Humber | 15.5 [13.4–17.5] | 12.4 [11.2–13.6] |  | 10.7 [9.4–12.0] | 11.2 [10.2–12.2] |  | | 605 [499–716] | | 507 [441–575] |
| East Midlands | 16.4 [14.1–18.7] | 13.7 [12.4–15.0] |  | 10.7 [9.2–12.2] | 10.8 [9.9–11.7] |  | | 641 [518–772] | | 540 [474–610] |
| West Midlands | 13.3 [11.4–15.2] | 13.4 [12.2–14.6] |  | 9.7 [8.6–10.8] | 10.9 [10.1–11.7] |  | | 471 [387–561] | | 533 [472–597] |
| East of England | 14.6 [12.8–16.5] | 13.9 [12.7–15.0] |  | 9.4 [8.2–10.7] | 11.8 [10.9–12.7] |  | | 501 [410–600] | | 599 [532–670] |
| London | 10.7 [9.4–12.0] | 15.2 [14.2–16.2] |  | 7.8 [6.8–8.8] | 8.6 [8.0–9.3] |  | | 305 [253–362] | | 477 [430–527] |
| South East | 14.4 [12.8–15.9] | 13.5 [12.5–14.4] |  | 9.3 [8.3–10.2] | 10.7 [10.0–11.4] |  | | 489 [420–564] | | 527 [479–579] |
| South West | 16.9 [14.6–19.1] | 14.3 [13.1–15.5] |  | 8.4 [7.4–9.4] | 10.0 [9.2–10.7] |  | | 518 [428–613] | | 522 [464–582] |
|  |  |  |  |  |  |  | |  | |  |

^1^ Proportion of adults who report currently smoking cigarettes (including hand-rolled) daily or non-daily.

^2^ Mean number of cigarettes smoked per adult per year. Calculated as: (mean daily cigarette consumption among adults who smoke cigarettes x proportion of adults who smoke cigarettes) x 365. 95% CIs were derived using Monte Carlo simulations.
